# ADM Scaffolds Generate a Pro-regenerative Microenvironment During Full-Thickness Cutaneous Wound Healing Through M2 Macrophage Polarization via Lamtor1

**DOI:** 10.3389/fphys.2018.00657

**Published:** 2018-06-04

**Authors:** Chengmin He, Zhi Yang, Ying Jin, Xiaoyang Qi, Jin Chu, Xiaoyuan Deng

**Affiliations:** MOE Key Laboratory of Laser Life Science, College of Biophotonics, South China Normal University, Guangzhou, China

**Keywords:** wound healing, ADM scaffolds, M2 macrophage, Lamtor1, skin regeneration

## Abstract

Adult mammalian skin has a defective regenerative capacity following full-thickness cutaneous injury; this defect overshadows the complete physiological functions of the skin. Immune-mediated skin reconstruction driven by biological scaffolds is a recently developed innovative repair strategy to support regenerative wound healing. However, to date, little is known about how biological scaffolds orchestrate the immune response to promote regeneration. Here, using acellular dermal matrix (ADM) scaffolds, we discovered that the default pro-inflammatory response was altered in response to a pro-regenerative response characterized by specific M2 polarization. M2 macrophages subsequently produced a series of wound healing factors, including matrix metalloproteinases (Mmps), and growth factors which promoted cell proliferation, stabilized angiogenesis, and remodeled the extracellular matrix. Our investigations further revealed that the M2 polarization of macrophages arose from an ADM scaffold-derived amino acid sufficiency signal by collagen degradation via macrophage phagocytosis, which activated the acid-sensing pathway (v-ATPase, Lamtor1, and mTORC1). Lamtor1, the acid-sensing pathway-associated lysosomal adaptor protein was critical for inducing M2 polarization, while with the presence of extracellular interleukin 4 (IL4). Our results suggest that ADM scaffolds generate a pro-regenerative microenvironment during full-thickness cutaneous wound healing through M2 macrophage polarization via Lamtor1.

## Introduction

Restoration of skin integrity and function following injury is crucial for maintaining the survival of most organisms because the skin performs multiple critical functions for the underlying organs. Skin-wound repair is a fundamental biological process that involves orchestrated cell signaling events and complex biochemical cascades. After damage, skin has a natural ability to undergo spontaneous repair and regeneration, but the capacity for regeneration is distinctly variable among different species and at different ages (Tanaka and Reddien, 2011). In the fetus, the skin has an extraordinary regenerative capacity and is capable of undergoing complete recreation following injury (Yates et al., 2012). However, in adult mammals, traumatic injuries generally result in excess fibrotic scar tissue and an absence of functional skin appendages (Takeo et al., 2015). Achieving scarless wound healing and functional restoration of damaged skin tissue in adults remains a great challenge.

The immune system is an active component of tissue repair and regeneration. Skin-wound healing begins with a local immune response characterized by massive recruitment of immune cells (Serhan et al., 2007) and their production of pro-inflammatory cytokines, such as TNF-α and IFN-γ (Falanga et al., 2002). Immune cells also secrete anti-inflammatory cytokines and growth factors to initiate the subsequent proliferative phase of healing and to promote the formation of vascularized granulation tissue with representative fibroblast-to-myofibroblast differentiation (triggered by TGF-β1; Hu and Phan, 2013) and the deposition of provisional extracellular matrix (ECM), which is the potential ultimate fibrous scar-converting tissue. Finally, the dermal tissue is remodeled, and the extending epidermis is reconstituted in parallel, leading to wound closure (Hinz et al., 2001).

The critical early event in immune response activation during injury determines whether the outcome of tissue restoration is positive remodeling or impaired repair with scar formation due to its heterogeneity (Godwin et al., 2017). Following injury, type 1 immunity, defined by the activity of neutrophils, type 1 innate lymphoid cells, T helper 1 cells (T_H_1 cells) and M1 macrophages, among others, supplies the primary function of instant defense against infection and clearance of necrotic tissue (Gieseck et al., 2017). However, persistent activity of this response can lead to chronic inflammation and even severe secondary damage, both of which prevent tissue regeneration (Forbes and Rosenthal, 2014). Similarly, certain cell types, such as type 2 innate lymphoid cells, T helper 2 cells (T_H_2 cells), and M2 macrophages, are thought to be associated with type 2 immunity, which is characterized by the secretion of anti-inflammatory cytokines and growth factors. Type 2 immunity has a verified role in establishing a regenerative microenvironment for effective cell replacement and restoring tissue structure and function (Forbes and Rosenthal, 2014). Nevertheless, type 2 immunity is also believed to be closely linked to fibrosis.

Among the various immune cell types, due to their versatility and plasticity, macrophages, and their derived phenotypes play a predominant role in the restoration of tissue homeostasis (Mosser and Edwards, 2008), which has been confirmed to be critical for regulating fibrosis and regeneration (Wynn and Barron, 2010). Indeed, macrophage depletion limits the continuity of the repair response, results in diminished wound debridement and leads to severe hemorrhage at the wound site (Zhang et al., 2012). Classically activated macrophages (M1 macrophages) enhance effective recruitment of defense components against pathogens, clear away cellular debris (Koh and DiPietro, 2011), and play a pivotal role in initiating angiogenesis (Spiller et al., 2014). Although their early roles are important, chronic M1 activation may prevent wound repair (Martin and Nunan, 2015). Dominant interleukin 4 (IL4)-activated M2 macrophages have been confirmed to be essential for wound repair due to their capacity to remodel the ECM and synthesize multiple cytokines and growth factors (such as Relmα, Egf, and Vegfα) (Sindrilaru et al., 2011), but uninterrupted M2 macrophage activation can also result in fibrosis (Wynn and Barron, 2010). Therefore, proper immune response regulation in wound healing is thought to be an active therapeutic target for manipulating the quality of the healing response toward reduced scar formation and improved tissue regeneration.

Regulating the immune response in wound healing via biological scaffolds has recently become an attractive regenerative strategy for directing tissue repair. Biological scaffolds provide a suitable niche to activate endogenous tissue repair (Willenborg et al., 2012). The impact of biological scaffolds on the immune system is thought to be the primary factor responsible for positive regeneration outcomes. The ability of scaffolds to promote regeneration by activating local macrophages toward an “M2” phenotype has been proposed by certain researchers (Chujo et al., 2009), but the mechanism responsible for this response has rarely been examined (Mimura et al., 2016). Understanding the immunomodulatory effect of biological scaffolds offers potential guidance for further desirable biomaterial development to improve tissue repair and regeneration. Unfortunately, to date, the immunomodulatory mechanism underlying the effects of biomaterial scaffolds on cutaneous wound healing has never been examined and remains largely unknown.

Acellular dermal matrix (ADM) consists of decellularized skin tissue obtained through removal of the cellular components and retention of the ECM structure. As a biological scaffold, it has demonstrated efficient improvement in skin reconstruction (Bondioli et al., 2014). The inflammatory response has long been known to be suppressed when macrophages engulf apoptotic cells (Sicari et al., 2012). Thus, intracellular nutrients, such as amino acids, that regulate macrophages have piqued our interest. In this study, we explore the possible underlying immunomodulatory mechanism of ADM scaffold-mediated promotion of full-thickness cutaneous wound healing. We show that during skin repair in mice, ADM scaffold-supported M2 polarization under IL4 conditions requires the lysosomal adaptor protein Lamtor1, which can be activated by biomaterial scaffold-derived amino acids. In addition, ADM scaffolds induce a pro-regenerative microenvironment through the production of numerous wound healing factors by M2 macrophages, including matrix metalloproteinases (Mmps) and growth factors, leading to a strong catalytic effect on skin reconstitution.

## Materials and methods

### Animals

Most specific pathogen-free BALB/c mice were purchased from the animal facility of Southern Medical University. Experiments were conducted in strict accordance with the Institutional Animal Care and Use Committee of the University of South China Normal University.

### Skin-wound modeling and ADM scaffold preparation

Skin-wound modeling (diameter: 7 mm) was performed as described previously (Wang et al., 2015). Full-thickness dorsal skin from neonatal and adult (12 weeks old) male BALB/c mice was used to prepare the ADM scaffolds. Dorsal hair was shaved, and the area was washed with water after the mice were euthanized by cervical dislocation. Full-thickness skin was harvested and washed in sterile phosphate-buffered saline (PBS) with 1% antibiotic-antimycotic (AA, Sigma) to produce the ADM scaffolds (Table 1).

**Table 1:** Reagents, concentrations, and time used for each decellularization procedure.

**Preparation of ADM Scaffolds**
Washed three times with PBS (with 1% AA)
0.25% Dispase^a^ in PBS (4°C, 48 h)
Separate the epidermis, leaving the dermal matrix
0.03% Triton in PBS, shaken (37°C, 2 × 24 h)
Wash with ddH_2_O, Ultrasonic oscillation (2 × 15 min)
Ethanol (25, 50, 75, and 100%)
Chloroform 1 min
Ethanol (100, 75, 50, and 25%)
Wash with PBS
Sterilize in 75% ethanol (12 h)
Washed with PBS (with 1% AA) before use

a *0.25% Dispase (Aoboxing, Beijing, China)*.

### TPEF-SHG imaging of ADM scaffolds

3D images of ADM scaffolds were acquired through the stack scan mode of TPLSCM (LSM 710 NLO coupled to a femtosecond Ti:sapphire laser; Zeiss, Jane, Germany) at 820 nm (collagen imaging). Simultaneously, the 3D image was scanned continuously (frame of a 2D image) for collagen at a speed of 10 s per frame.

### Sem imaging of ADM scaffolds

The ADM scaffolds were fixed in cold 2.5% glutaraldehyde (4°C, 24 h) and then dehydrated by immersion in graded ethanol solutions (30, 50, 75, 85, and 95%; each for 15 min), followed by immersion in hexamethylene diamine (3 × 15 min) and subsequent air drying. The dried ADM scaffolds were sputter-coated with a 30-nm gold layer for SEM (Zeiss Ultra 55, Carl Zeiss, Jena, Germany).

### Histological and immunofluorescence analysis

Wound samples (diameter: 7 mm) were sectioned perpendicularly into 10-μm-thick paraffin sections after fixation in 4% polyformaldehyde at 4°C for 24 h. To assess wound tissue, 4–6 sections spanning the wound sample per mice were stained with H&E. Immunofluorescence staining was performed to quantify skin regeneration. The following primary antibodies were used: rabbit polyclonal anti-F4/80 (1:100, Abcam), rabbit polyclonal anti-CD86 (1:200, Abcam), rabbit polyclonal anti-CD206 (1:200, Abcam), rabbit polyclonal anti-Ki67 (1:100, Cell signaling), and rabbit polyclonal anti-CD31 (1:300, Abcam). Immune complexes were visualized with a FITC-conjugated secondary antibody (1:100, Santa Cruz). Nuclei were labeled with DAPI (Sigma) for 15 min. Images were acquired with a Zeiss LSM 710 confocal microscope (Carl Zeiss) or an AxioM1 light microscope (Carl Zeiss). The fluorescence intensity and cell number were acquired and analyzed using Image-pro plus.

### Cell culture and SiRNA transfection

Mouse macrophages (RAW 264.7, BNCC) were cultured in 90% DMEM (Gibco) + 10% FBS (fetal bovine serum, Gibco) supplemented with 100 U/mL penicillin/streptomycin. siLamtor1(Sense: 5′-GCGAAAGAAGAGCUGGUUGTT-3′; Antisense: 5′-CAACCAGCUCUUCUUUCGCTT-3′) and siNC (Sense: 5′-UUCUCCGAACGUGUCACGUTT-3′; Antisense: 5′-ACGUGACACGUUCGGAGAATT-3′) were purchased from Sangon Biotech (Shanghai, China) and transfected into macrophages with Lipofectamine (Thermo Fish Scientific, USA). Seeded cells that had reached 60–80% confluence in 6-well plates, 6-well plates covered with ADM, or 6-well plates treated with collagen (1 mg/ml) were then incubated with siLamtor1-lipid complex (siLamtor1 concentration of 25 pmol/well) or an equal volume of siMock-lipid complex for 12 h.

### Macrophage activation and flow cytometry

Macrophages were stimulated with IL4 (50 ng/ml, PeproTech, USA) for 24 or 48 h and then harvested and washed with cold 1 × PBS. Intracellular labeling with anti-mouse CD206-PE (BD Biosciences, USA) was performed using the Intracellular FIX&PERM Reagent set from MULTISCIENCES (China) according to the manufacturer's protocol. Cytofluorometric data were acquired with a BD FACS Canto II flow cytometer and analyzed using FlowJo 10.0.7.

### qRT-PCR analysis

Total RNA from wound tissue and single cell suspensions were isolated with TRIzol reagent (TaKaRa, China). cDNA synthesis was performed using a PrimeScript^TM^ RT reagent Kit with gDNA Eraser (TaKaRa, China). Primers were synthesized by Sangon Biotech (China) and are described in Table 2. qRT-PCR was conducted on a TaKaRa Real Time PCR Machine using SYBR Green (SYBR® *Premix Ex Taq*^TM^, TaKaRa, China) as a reporter and validated with a 7500 Real Time PCR system (Applied Biosystems). The expression level of the target gene normalized to *Act*β expression was calculated using the comparative method of relative quantification (2^−ΔΔ^*C*_t_).

**Table 2:** Primer sequences.

**Gene**	**Forward primer (5′->3′)**	**Reverse primer (5′->3′)**
*Tnfα*	AGGTTCTCTTCAAGGGACAA	CCTGGTATGAGATAGCAAATCG
*iNOS*	CCTATCTCCATTCTACTACTACCA	ACCACTTTCACCAAGACTCTA
*Relmα*	TACTGGGTGTGCTTGTGGCTTTGC	GGCAGTGGTCCAGTCAACGAGTAAG
*Arg1*	GAAGAATGGAAGAGTCAGTGTG	GGAGTGTTGATGTCAGTGTG
*Jag2*	GTGTGGTTATCTGCGTATGG	GTTGCGGATGGGATTGAG
*IL4*	CTAGTTGTCATCCTGCTCTTCT	CTTCTCCTGTGACCTCGTTC
*Ifnγ*	ATGAACGCTACACACTGC	CCACATCTATGCCACTTGAG
*Tbx21*	CGCATCTGTTGATACGAGTG	TGGTTGGATAGAAGAGGTGAG
*Lamtor1*	CAACTACCATAGCCTACCTTCA	GTCCATGTACTCATGCTGTTC
*Mmp3*	ATGGTATTCAGTCCCTCTATGG	TGGTGATGTCTCAGGTTCC
*Mmp9*	ACTACGATAAGGACGGCAA	TCAAAGATGAACGGGAACAC
*Egf*	GAATATCGGTGCTGACTCTG	TGCTTGATGCCTGATAAGAC
*Igf*	TTTACTTCAACAAGCCCACAG	GAAGCAACACTCATCCACAA
*Vegfα*	GCCTTGTTCAGAGCGGAGAA	CCTTGGCTTGTCACATCTGC
*Tgfβ*	CTGCTGACCCCCACTGATAC	AGCCCTGTATTCCGTCTCCT
*Actβ*	CGTTGACATCCGTAAAGACC	TAGGAGCCAGAGCAGTAATC

### Statistical analyses

Statistical analyses were performed with GraphPad Prism 7.0 for Windows, and differences among the 3 groups were evaluated with one-way ANOVA followed by Dunnett's multiple comparison test. Statistical significance levels, denoted by a single asterisk (*P* < 0.05), two asterisks (*P* < 0.01), or three asterisks (*P* < 0.001), are indicated in each figure.

## Results

### ADM scaffolds induce recruitment of macrophages in skin-wound healing

**Figure 1:**
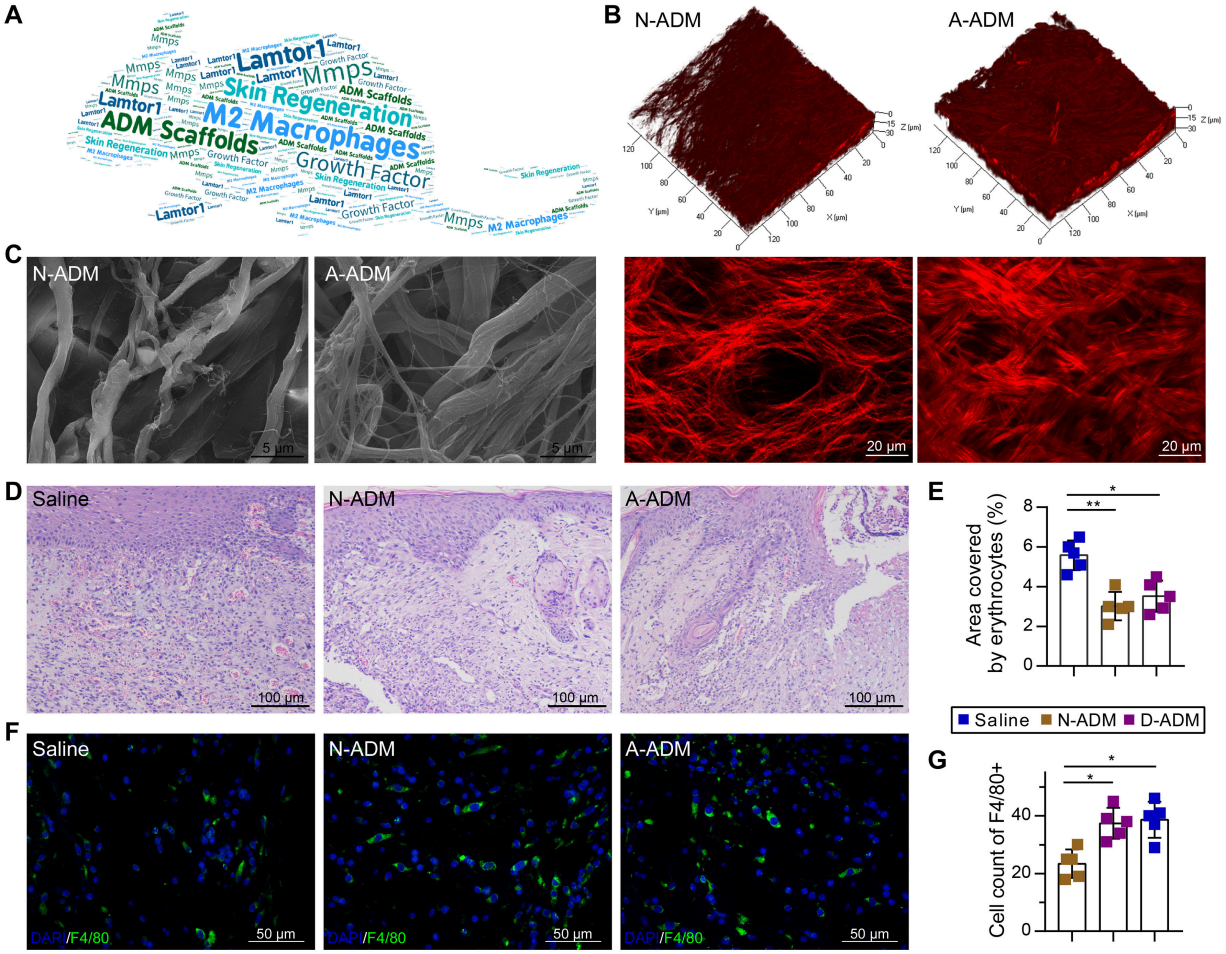
ADM scaffolds induce recruitment of macrophages in skin-wound healing. **(A)** Word map showing key objectives of the study. **(B)** TPF/SHG 3D scanning images of N-ADM and A-ADM scaffolds (up); 2D images of N-ADM and A-ADM scaffolds (down). **(C)** Ultrastructural images of collagen fibrils in N-ADM and A-ADM scaffolds. **(D)** Representative H&E staining of wounds at 10 days after injury. **(E)** Quantification of the hemorrhage area in wound tissue (*n* = 5 from two experiments). **(F)** Representative micrographs showing immunostaining of macrophages with F4/80 at 10 days after injury. **(G)** Quantification of F4/80-positive cells in wound tissue (*n* = 5 mice from two experiments). ANOVA: ^*^P < 0.05, ^**^P < 0.01, ^***^P < 0.001.

To investigate the role of ADM scaffolds in skin repair, scaffolds were prepared through degradation of neonatal and adult mouse skin, and full-thickness excision skin wounds were inflicted on the backs of adult mice. To ensure a similar amount of collagen in both scaffolds, we transplanted two-layer neonatal mouse skin ADM (N-ADM) scaffolds and single-layer adult mouse skin ADM (A-ADM) scaffolds in skin wounds. Dynamic optical two-photon fluorescence and second-harmonic generation (SHG) and scanning electron microscopy (SEM) images revealed the shape, orientation, and density of collagen fibers and the spacious structural characteristics of ADM scaffolds (Figures 1B,C).

Morphological analysis via hematoxylin and eosin (H&E) staining of wound tissue sections at 10 days after injury revealed major differences in the repair response in the saline-treated mice versus the ADM scaffold-transplanted mice. Whereas the ADM scaffold-transplanted mice developed vascularized and cellular wound tissue by 10 days post-injury, in the saline-treated mice, the wound tissue was highly hemorrhagic (1.56- and 1.59-fold increase in the area encompassed by erythrocytes in the saline-treated mice compared with the area in the N-ADM and A-ADM mice, respectively), indicative of defective repair response (Figures 1D,E), which could be due to the absence of macrophages (Lucas et al., 2010).

To examine whether ADM scaffolds promote the recruitment of macrophages to infiltrate the wound site, we stained wound tissue sections with F4/80 antibody and subjected them to quantitative analysis. The number of macrophages recruited to the skin injury site was dynamically increased in the N-ADM and A-ADM mice compared with that in the saline-treated mice, peaking with a 1.8- and 1.9-fold increase, respectively (Figures 1F,G).

### ADM scaffolds accelerate M2 polarization in skin-wound healing

**Figure 2:**
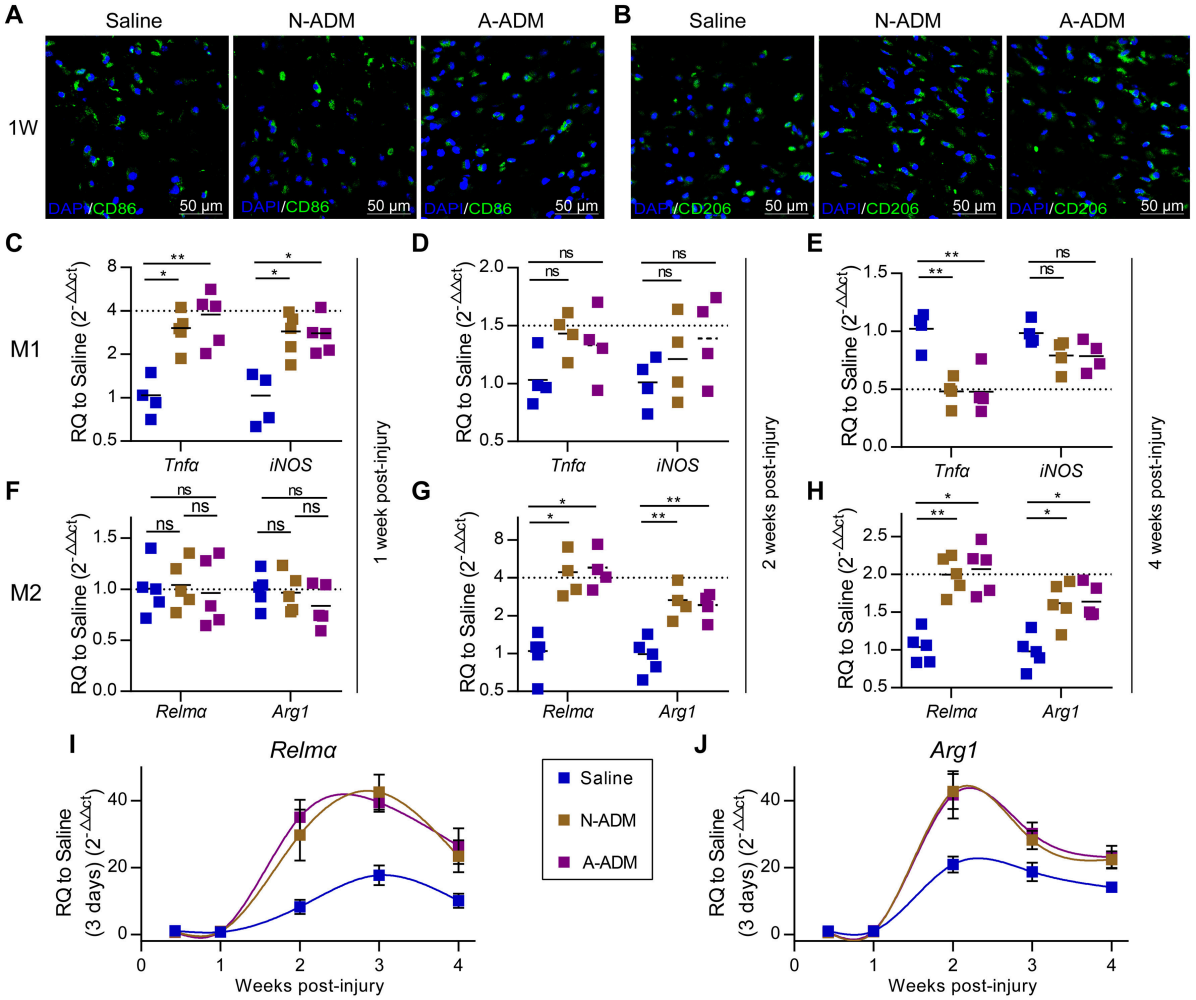
ADM scaffolds induce M2 polarization in skin-wound healing. **(A)** Representative micrographs showing immunostaining of macrophages with CD86 at 1 weeks after injury. **(B)** Representative micrographs showing immunostaining of macrophages with CD206 at 1 weeks after injury. **(C–E)** qRT-PCR analysis of M1 signature gene (*Tnf*α and *iNOS*) expression displayed as the fold change compared with the saline control at **(C)** 1 week after injury, **(D)** 2 weeks after injury, and **(E)** 4 weeks after injury (*n* = 4–5 mice from two experiments). **(F–H)** qRT-PCR analysis of M2 signature gene (*Relm*α and *Arg1*) expression displayed as a fold change over saline control at **(F)** 1 week after injury, **(G)** 2 weeks after injury, and **(H)** 4 weeks after injury (*n* = 4–5 mice from two experiments). **(I,J)** M2 signature gene expression (qRT-PCR) of **(I)** *Jag2* and **(J)** *IL4* displayed as the fold change compared with the saline control (3 days post-injury) from 3 days to 4 weeks (*n* = 4–5 mice from two experiments). ANOVA: ^*^P < 0.05, ^**^P < 0.01, ^***^P < 0.001.

Macrophages grown from bone marrow were unstimulated (M0) or activated (M1 and M2). Next, we sought to identify the types of macrophage cell population dynamics compromised in the saline-treated mice. As a prelude, we assessed M1 and M2 cell polarization in the wound sites. Adult mice were treated with saline, N-ADM scaffolds and A-ADM scaffolds in the wound sites, and the healing skin tissue sections were analyzed via immunofluorescence over a 7-day time course at 1 weeks after injury. Immunolabeling studies revealed a more accumulation of CD86^+^ cells (a costimulatory molecule expressed at high levels by classical M1 macrophages) and a similar accumulation of CD206^+^ cells (a mannose receptor and classical M2 marker) in ADM scaffold-treated mice compared with this in saline-treated mice (Figures 2A,B), indicating that the ADM scaffolds can regulate macrophage polarization.

To further elucidate the impact of the ADM scaffolds on macrophages, we evaluated the expression of classic M1 signature genes, such as *Tnf*α and *iNOS*, and classic M2 signature genes, such as *Relm*α and *Arg1*. At 1 week after implantation, the presence of scaffolds in damaged skin significantly increased the expression of *Tnf*α and *iNOS*, which peaked with a 3- to 4-fold increase over that in the saline-treated mice (Figure 2C); however, the expression of M2 signature genes (*Relm*α and *Arg1*) was similar between the N-ADM scaffold-treated and the saline-treated mice (Figure 2F). Taken together, these findings indicated that the mice had rejected the transplanted scaffolds.

However, in the ADM scaffold-treated mice, up-regulation of M1 signature gene expression was mitigated at 2 weeks after injury, with decreased expression at 4 weeks (Figures 2D,E). In contrast, ADM scaffolds showed increased expression of genes associated with M2 polarization, including *Relm*α and *Arg1* (Figures 2G,H). More specifically, the expression of *Relm*α peaked with an increase of more than 4-fold over that in the saline mice at 2 weeks and 2-fold at 4 weeks, and the expression of *Arg1* simultaneously increased more than 2-fold and 1.5-fold at 2 and 4 weeks, respectively.

To demonstrate in detail the altered tendency of M2 macrophages during diverse healing stages, we analyzed the relative expression of M2 signature genes (*Relm*α and *Arg1*) and compared the results to those obtained for the saline-treated mice on day 3 (Figures 2I,J). During the early stage of repair (3–7 days post-injury), *Relm*α and *Arg1* mRNA expression was barely detectable. Conversely, qRT-PCR analysis showed that the wounds in the three groups of mice showed a dynamic increase in *Relm*α and *Arg1* expression during the middle (2–3 weeks post-injury) and late (4 weeks post-injury) stages of repair. Simultaneously, a difference was already apparent at 1–2 weeks in the ADM scaffold-treated mice compared with the saline-treated mice, with either *Relm*α or *Arg1* peaking with a highly significant increase (*Relm*α: an ~4-fold increase compared with the saline-treated mice at 2 weeks and an ~2-fold increase at 3 and 4 weeks, Figure 2I; *Arg1*: an ~2-fold increase compared with the saline-treated mice at 2 weeks and an ~1.5-fold increase at 3 and 4 weeks, Figure 2J).

### Collagen is crucial for M2 polarization in skin wounds

**Figure 3:**
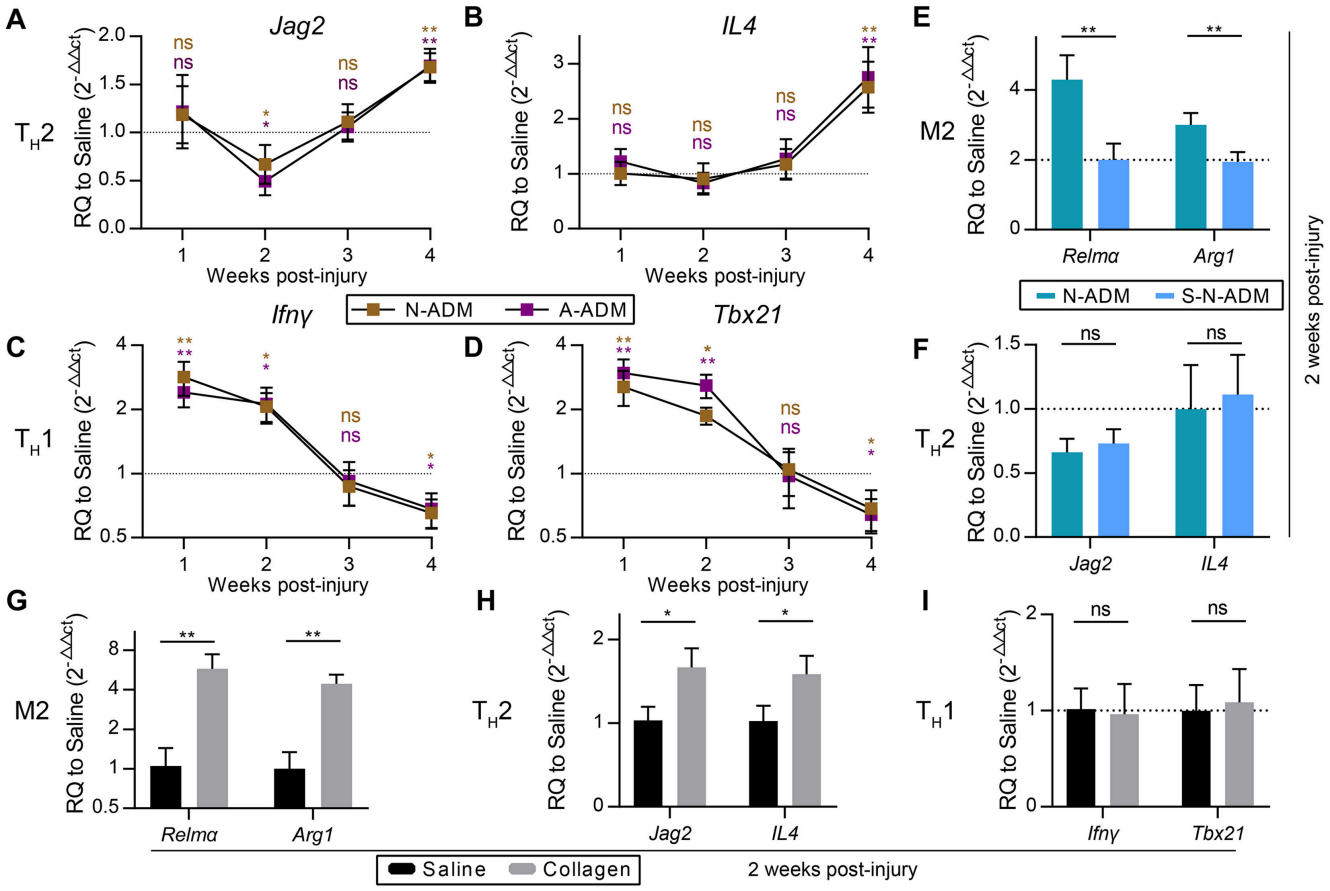
M2 pro-regenerative myeloid polarization accelerated by scaffolds is not directly dependent on T_H_2 cells. **(A,B)** T_H_2 signature genes expression (qRT-PCR) of **(A)** *Jag2* and **(B)** *IL4* displayed as a fold change over saline control at indicated weeks (*n* = 4–6 mice from two experiments). **(C,D)** T_H_1 signature genes expression (qRT-PCR) of **(C)** *Ifn*γ and **(D)** *Tbx21* displayed as a fold change over saline control at indicated weeks (*n* = 4–6 mice from two experiments). **(E)** M2 signature genes expression (qRT-PCR) of *Relm*α and *Arg1* displayed as a fold change over saline control at 2 weeks after wound treatment with N-ADM and single layer of N-ADM (S-N-ADM) (*n* = 4 mice from two experiments). **(F)** T_H_2 signature genes expression (qRT-PCR) of *Jag2* and *IL4* displayed as a fold change over saline control at 2 weeks (*n* = 4 mice from two experiments). **(G)** M2 signature genes expression (qRT-PCR) of *Relm*α and *Arg1* displayed as a fold change over saline control at 2 weeks after wound treatment with collagen (*n* = 4 mice from two experiments). **(H)** T_H_2 signature genes expression (Real-time PCR) of *Jag2* and *IL4* displayed as a fold change over saline control at 2 weeks (*n* = 4 mice from two experiments). **(I)** T_H_1 signature genes expression (qRT-PCR) of *Ifn*γ and *Tbx21* displayed as a fold change over saline control at 2 weeks (*n* = 4 mice from two experiments). ANOVA **(A–F)** and Student's *t-*test **(G–I)**: ^*^P < 0.05, ^**^P < 0.01, ^***^P < 0.001.

We next sought to explore the mechanisms underlying M2 polarization during the middle and late phases of wound healing. The expression of *IL4*, a gene encoding a canonical type 2 helper T cell (T_H_2) cytokine and regarded as the predominant cause of M2 polarization, was examined in these experiments. Unexpectedly, the expression of *IL4* was not significantly different between the N-ADM scaffold-treated, A-ADM scaffold-treated and saline-treated mice during the early and middle phases of repair (Figure 3B), and similar results were obtained for *Jag2* (Figure 3A), which encodes the Notch ligand Jagged 2 that helps direct T_H_ differentiation away from T_H_1 and toward T_H_2 (Fang et al., 2007). Conversely, the scaffolds induced a T_H_1-type gene expression profile characterized by increased expression of *Ifn*γ and *Tbx21* (T_H_1 canonical genes) for more than 2 weeks after injury. Our findings suggest that M2 polarization is not directly dependent on T_H_2 cells.

To test whether M2 polarization in affected regions was associated with the amount of scaffold, we transplanted single-layer N-ADM (S-N-ADM) scaffolds into traumatic skin wounds of mice. Notably, the presence of S-N-ADM scaffolds decreased the expression of M2 signature genes at 2 weeks after injury (*Relm*α: 0.52-fold decrease compared with N-ADM mice, Figure 3E; *Arg1*: ~0.67-fold decrease compared with N-ADM mice, Figure 3E). In addition, T_H_2-associated genes (*Jag2* and *IL4*) were not up-regulated in S-N-ADM scaffold-treated mice (Figure 3F). This finding suggests that the amount of scaffold material may affect M2 polarization. We also noticed that scaffolds increased *Relm*α and *Arg1* expression from 2 to 4 weeks (Figures 2I,G), after which the expression of *Jag2* and *IL4* were up-regulated and *Ifn*γ and *Tbx21* were down-regulated at 4 weeks (Figures 3A,B). Thus, the relationship between M2 and T_H_2 may be promoted, during which the polarization of M2 might drive T_H_2 differentiation, but the relationship between M2 and T_H_1 is opposite.

In the early stages of scaffold transplantation, M1 signature genes sharply increased but showed a rapid regression after 2 weeks with degradation of the scaffolds (Figures 2C–E). We hypothesized that collagen, the major component of the scaffolds, was the fundamental cause of ADM scaffold support of M2 polarization. As expected, collagen-treated wounds showed the higher expression of M2 signature genes (*Relm*α and *Arg1*) compared with saline-treated mice at 2 weeks after injury (Figure 3G). In addition, collagen promoted an increase in the T_H_2 response (*Jag2*: 1.68-fold increase compared with the saline-treated mice, Figure 3H; *IL4*: 1.54-fold increase compared with the saline-treated mice, Figure 3H), but T_H_1 responses were similar to those observed in the saline-treated mice (Figure 3I).

**Figure 4:**
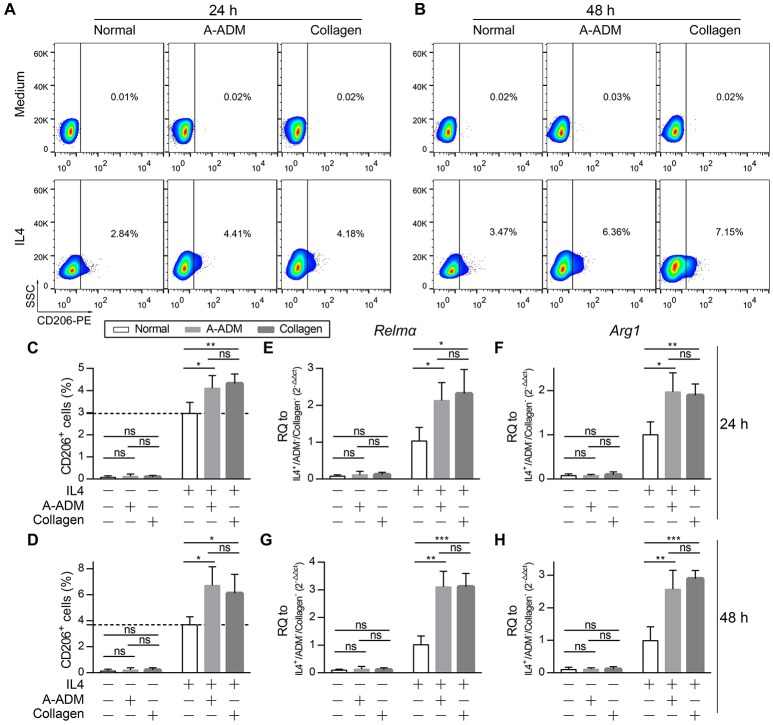
Collagen accelerate M2 polarization *in vitro* with similar amounts of IL4. **(A,B)** *In vitro* flow cytometric analysis of macrophage (RAW 264.7) cell line treat with A-ADM or collagen (1 mg/ml) concurrently stimulated by 50 ng/ml IL4 (*n* = 4 from two experiment). CD206 = type-2 alternative macrophage. **(C,D)** Summary data for fraction of CD206^+^ macrophages. **(E,F)** M2 signature genes expression (qRT-PCR) of *Relm*α **(E)** and *Arg1* **(F)** displayed as a fold change over control at 24 h after cell culture with A-ADM and collagen (*n* = 4 from two experiments). **(G,H)** M2 signature genes expression (qRT-PCR) of *Relm*α **(G)** and *Arg1* **(H)** displayed as a fold change over control at 48 h (*n* = 5 from two experiments). ANOVA: ^*^P < 0.05, ^**^P < 0.01, ^***^P < 0.001.

For comparison, M2 macrophages that were classically activated with an equal amount of IL4 (50 ng/ml) under diverse incubation circumstances, including the presence of A-ADM scaffold and collagen (1 mg/ml), were also examined *in vitro. In vitro* flow cytometric analysis of M2 macrophages (CD206^+^ cells; definition of the positive gates is described in the Methods) showed that both A-ADM scaffolds and collagen increased M2 polarization that was induced by an equal amount of IL4 (Figures 4A,B). Specifically, culturing the macrophage cell line on A-ADM^IL4+^ scaffolds induced a 1.33- and 1.84-fold increase in M2 polarization over the normal^IL4+^ at 24 and 48 h, respectively (Figures 4C,D). Similarly, culturing the macrophage cell line with collagen^IL4+^ induced a 1.42- and 1.61-fold increase in M2 polarization over the normal^IL4+^ at 24 and 48 h, respectively (Figures 4C,D). We further verified M2 polarization using qRT-PCR to compare the *Relm*α and *Arg1* gene expression between different treatments. As expected, M2 activation, as measured by increased *Relm*α and *Arg1* expression, was detected in the A-ADM^IL4+^- and collagen^IL4+^-treated macrophages when compared with that in the normal^IL4+^ macrophages (Figures 4E–H). These data indicated that the M2 response also had a collagen-independent role in shaping the wound healing response.

### Lamtor1 is essential for collagen-mediated induction of M2 polarization

An obvious question was which factor(s) might mediate collagen induction of M2 polarization after traumatic skin injury. Our attention was rapidly drawn to Lamtor1, the amino acid-sensing pathway (v-ATPase, Lamtor1, and mTORC1)-associated lysosomal adaptor protein, which is indispensable for inducing M2 polarization along with the presence of extracellular IL4 and amino acids (Kimura et al., 2016).

**Figure 5:**
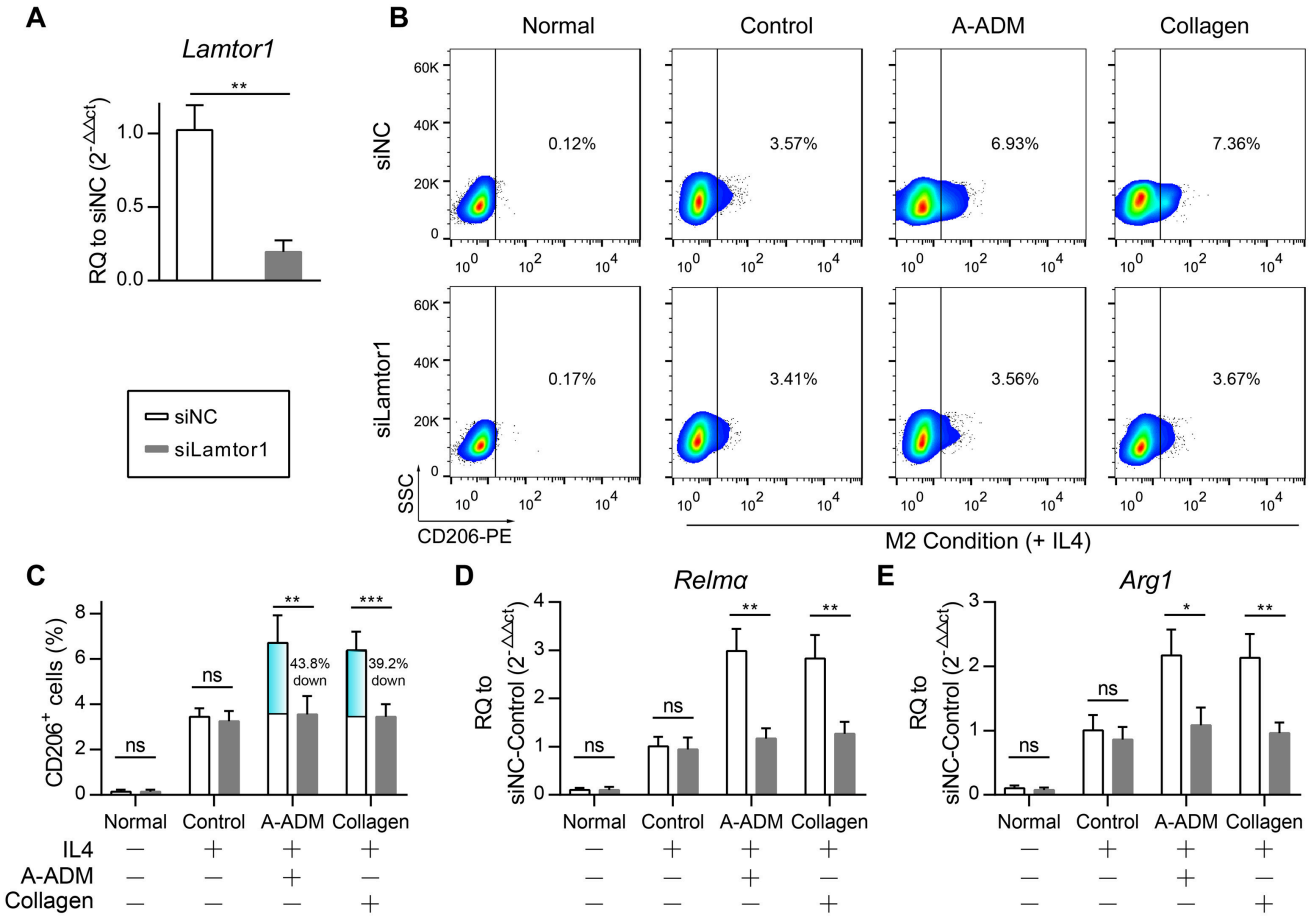
Lamtor1 is essential for ADM scaffold induction of M2 polarization *in vivo*. **(A)** qRT-PCR for Lamtor1 mRNA confirmed successful knock-down of Lamtor1 mRNA in macrophages. **(B)** *In vitro* flow cytometric analysis of Lamtor1-deficient macrophages treated with A-ADM or Collagen (1 mg/ml) concurrently stimulated by 50 ng/ml IL4 (*n* = 4 from two experiment). CD206 = type-2 alternative macrophage. **(C)** Summary data for fraction of CD206^+^ macrophages. **(D,E)** M2 signature genes expression (qRT-PCR) of *Relm*α **(D)** and *Arg1* **(E)** displayed as a fold change over control at 48 h after Lamtor1-deficient macrophages treated with A-ADM and Collagen (*n* = 4 from two experiments). Student's *t-*test **(A,C–E)**: ^*^P < 0.05, ^**^P < 0.01, ^***^P < 0.001.

To investigate the role of Lamtor1 in macrophages, we established a knockdown (siLamtor1) cell line lacking Lamtor1 in macrophages. qRT-PCR analysis of the siLamtor1-macrophages confirmed efficient *Lamtor1* suppression (0.21-fold reduction compared with siNC-macrophages, Figure 5A). M2 macrophages (CD206^+^ cells) were counted via flow cytometry, and the percentage of M2 macrophages among all macrophages revealed that the increase in CD206^+^ cells was abrogated in both the ADM^IL4+^ scaffold- and collagen^IL4+^-treated siLamtor1-macrophages (Figures 5B,C). As observed for CD206^+^ cells, the induction of these M2 signature genes were almost completely lost in the siLamtor1-macrophages (Figures 5D,E). The macrophages cultured on A-ADM^IL4+^ scaffolds or with collagen^IL4+^ and treated with siLamtor1 both produced >2-fold less *Relm*α and >1.5-fold less *Arg1*, indicating that the lysosomal adaptor protein Lamtor1 was essential for scaffold- or collagen-induced M2 polarization.

### M2 responses to ADM scaffold-treated skin wounds promote functional tissue regeneration

**Figure 6:**
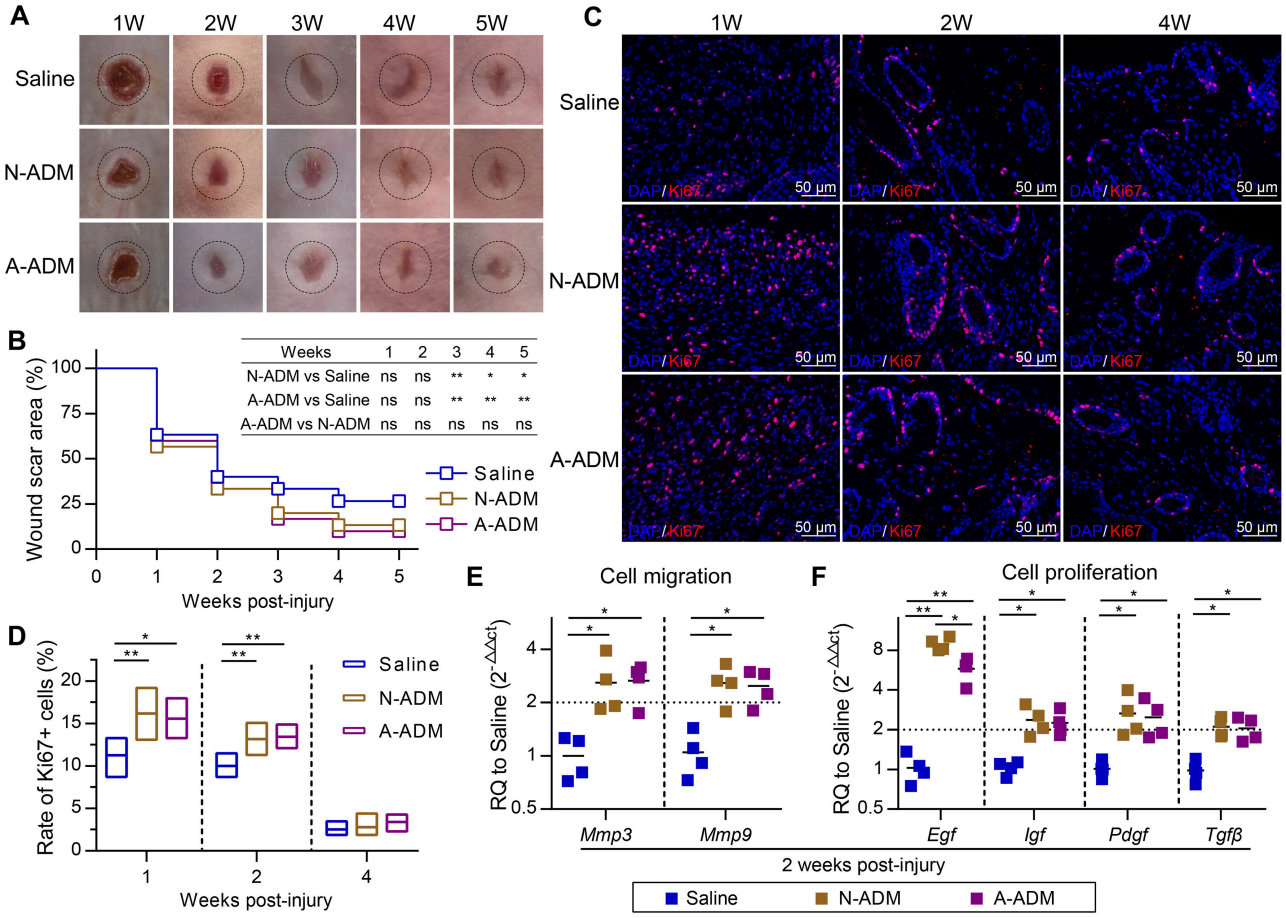
ADM scaffolds promote cell proliferation and migration during skin-wound healing. **(A)** Representative photographs showing the wounds after treatment with N-ADM, A-ADM, or Saline at indicated weeks after injury. **(B)** Quantification of scar percentage in wounds (*n* = 5 mice from two experiments). **(C)** Representative micrographs showing the immunostaining of proliferation cells with Ki67 at indicated weeks after injury. **(D)** Quantification the rate of Ki67-positive cells in wounds (*n* = 5 mice from two experiments). **(E)** qRT-PCR analysis of cell migration signature genes (*Mmp3* and *Mmp9*) expression displayed as a fold change over saline control at 2 weeks after injury (*n* = 5 mice from two experiments). **(F)** qRT-PCR analysis of cell proliferation signature genes (*Egf*, *Igf*, *Tgf*β, and *Pdgf*) expression displayed as a fold change over saline control at 2 weeks after injury (*n* = 4–5 mice from two experiments). ANOVA: ^*^P < 0.05, ^**^P < 0.01, ^***^P < 0.001.

Subcutaneous ADM scaffold implants produce an M2 response in wounds, but the connection to wound healing and regeneration is unknown. Recent studies have indicated that macrophages with the favorable M2 phenotype encourage constructive tissue remodeling due to their capacity to remodel the ECM and synthesize multiple cytokines and growth factors (Wynn and Vannella, 2016). Consistent with this finding, we observed that the scaffolds increased the expression of genes associated with cell proliferation and migration. More specifically, *Mmp3* and *Mmp9* mRNA, encoding proteins that regulate cell migration, were induced >2-fold more than those in the saline-treated mice (Figure 6E), which suggested that the high level of Mmps promoted remodeling of the ECM at the wound front, allowing the front cells to progress toward the center and leading to rapid wound closure (Figures 6A,B). In addition, qRT-PCR analysis of the complete wound tissue also revealed up-regulation of *Egf*, *Igf*, *Pdgf*, and *Tgf*β, even *Egf* expression was up-regulated >6-fold in comparison to its expression in the saline-treated wounds (Figure 6F); these genes are linked to cell proliferation and differentiation. The cells in the wound showed increased expression of the cell proliferation marker Ki67, supporting the enhanced ability of ADM scaffolds to promote wound regeneration (Figures 6C,D).

**Figure 7:**
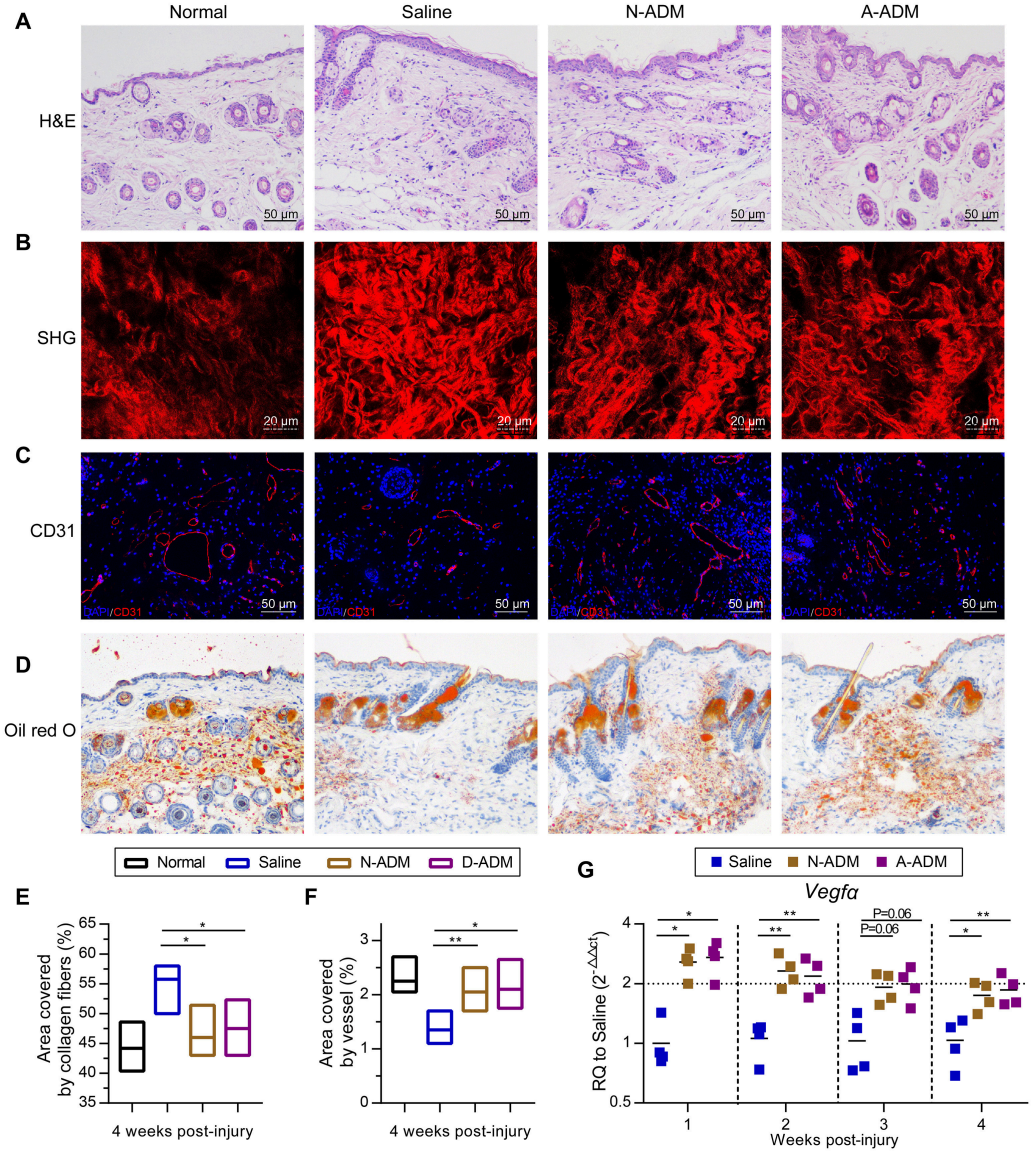
Regeneration and reconstruction of skin structures after ADM scaffold treatment. **(A)** Representative H&E staining of new skin tissue at 4 weeks after injury. **(B)** TPF/SHG scanning images of new skin tissue at 4 weeks after injury. **(C)** Representative micrographs showing the immunostaining of vessel area with CD31 at 4 weeks after injury. **(D)** Representative oil red o staining of new skin tissue at 4 weeks after injury. **(E)** Quantification of collagen fibers area in wound sites of four groups based on TPF/SHG scanning images (*n* = 5 mice from two experiments). **(F)** Quantification of vessel area in wound sites of four groups based on CD31 staining (*n* = 5 mice from two experiments). **(G)** *Vegf*α displayed as a fold change over saline control at indicated weeks after wound treatment with N-ADM, or A-ADM (*n* = 5 mice from two experiments). ANOVA: ^*^P < 0.05, ^**^P < 0.01, ^***^P < 0.001.

To gain further insights into the healing outcome, we performed H&E staining of new skin tissue 4 weeks after injury. Newly generated hair follicles were observed only in the N-ADM and A-ADM group and were absent in the saline-treated wound (Figure 7A). Collagen structures in the dermis of regenerated skin in the three groups at 4 weeks were visualized with TPF/SHG scanning images. Collagen fibers in the N-ADM and A-ADM groups were more similar to normal dermal tissue, with a reduced content, and more scattered fibers, than those in the saline-treated wounds (Figures 7B,E). Interestingly, although wound healing in adult mice generally results in a scar with the absence of subcutaneous fat, we found that ADM scaffolds promoted subcutaneous fat accumulation (Figure 7D). These data indicated that the healing dynamics were accelerated by ADM scaffolds.

The angiogenic response, which is an important step in the wound healing process, was also assessed. Immunohistology confirmed that vascular networks and a large number of endothelial cells (CD31^+^ cells) were present in and around the wounds treated with ADM scaffolds (>2% in scaffold-treated versus ~1.4% in saline-treated wounds) at 4 weeks after injury (Figures 7C,F). These findings were subsequently confirmed by qRT-PCR analysis. Indeed, the ADM scaffolds induced an ~2-fold increase in *Vegf*α expression over the saline-treated wounds from 1 to 4 weeks after injury (Figure 7G).

## Discussion

**Figure 8:**
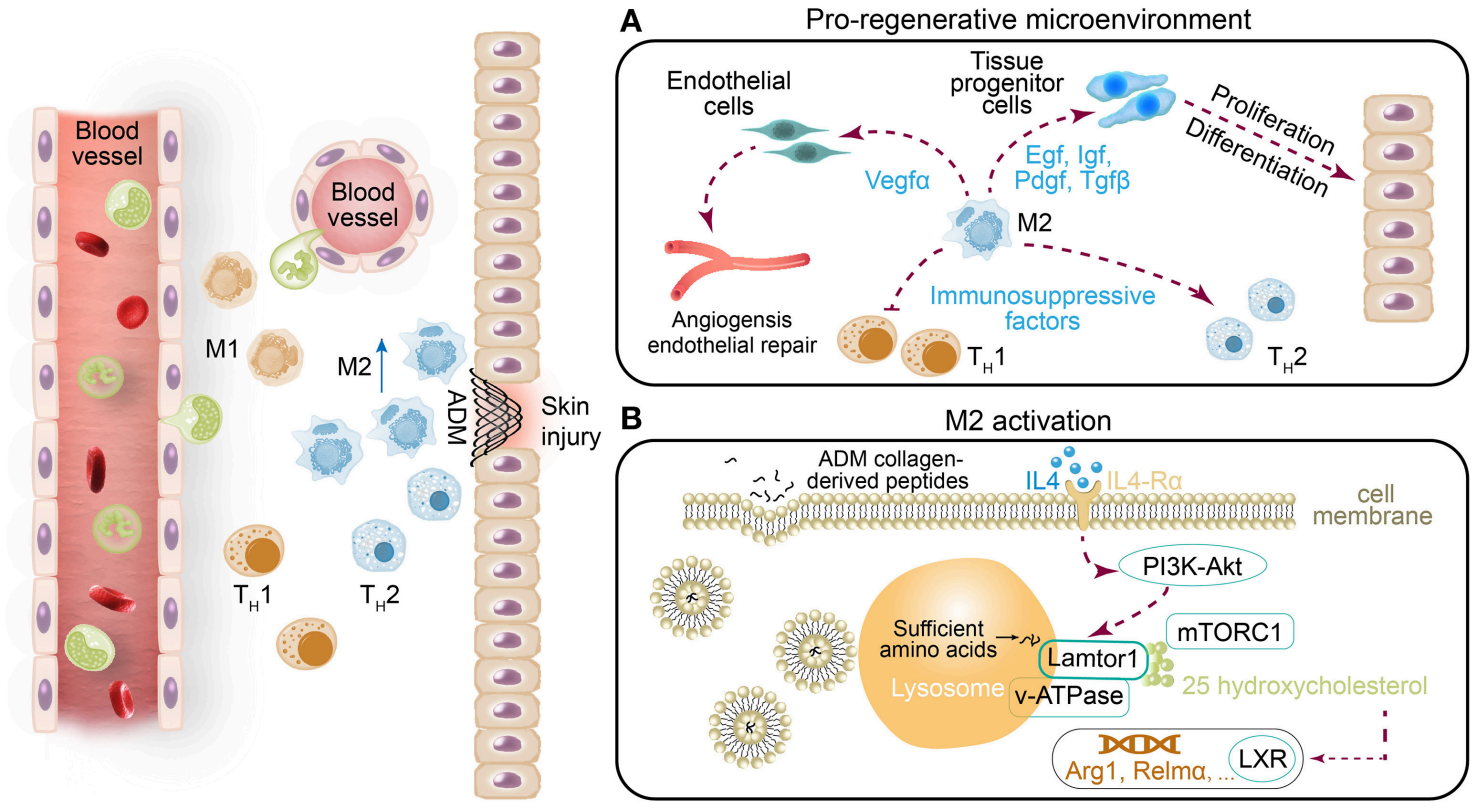
**(A)** In addition to the previously identified ADM scaffold-induced change from the default pro-inflammatory response to a pro-regenerative response via M2 macrophage polarization, **(B)** we found that Lamtor1, the amino acid-sensing pathway (v-ATPase, Lamtor1, and mTORC1)-associated lysosomal adaptor protein (Kimura et al., 2016), is indispensable for ADM scaffold-mediated induction of M2 macrophage polarization.

In this study, we used a mouse skin injury model and two types of ADM scaffolds to explore the cellular and molecular response to implantation. We found that the scaffolds generated a pro-regenerative microenvironment through abundant production of wound healing factors by M2 macrophages, such as Mmps (Mmp3 and Mmp9) and a series of growth factors (Egf, Igf, Pdgf, Tgfβ, and Vegfα), which promoted cell proliferation, stabilized angiogenesis, and remodeled the extracellular matrix (Figure 8A). Our investigation further revealed that the M2 polarization of macrophages arose from amino acid sufficiency signal, derived from collagen degradation of ADM scaffolds via phagocytosis of macrophage, which activated the acid-sensing pathway (v-ATPase, Lamtor1 and mTORC1) (Figure 8B) (Kimura et al., 2016). The acid-sensing pathway-associated lysosomal adaptor protein, Lamtor1, was essential for the process of ADM scaffold-induced M2 polarization, while with the presence of IL4. Collectively, the ADM scaffolds changed the default pro-inflammatory response to a pro-regenerative response through M2 polarization via Lamtor1.

The conversion of macrophages from M0/M1 to M2 is believed to be important for faultless wound repair (Brancato and Albina, 2011; Brown et al., 2012). Brown et al. demonstrated that an increased number of M2 macrophages and a higher ratio of M2:M1 macrophages led to more positive remodeling outcomes (Brancato and Albina, 2011). Modulation of the inflammatory response through induction of M2 polarization has become a strategy for biomaterial assessment in regenerative medicine. Our experiments suggested that the immunoregulatory effect of the ADM scaffold on M2 polarization in cutaneous wound healing occurred via the amino acid-sensing pathway in macrophages. Digestion of phagocytosed cells by macrophages provides continuous abundant production of nutrients such as amino acids for macrophages. This activity is vital for promoting M2 polarization and suppressing the inflammatory response (Voll et al., 1997; Maderna and Godson, 2003; Chantranupong et al., 2015). Mmps are well known to degrade a variety of ECM proteins into peptides. Two types of *Mmps, Mmp3*, and *Mmp9*, were found to be abundantly expressed in ADM-treated wounds (Figure 6E). Phagocytosis of peptides by macrophages is thus considered to provide a sufficient amino acid source in lysosomes for macrophages. In the presence of sufficient nutrients in cells and an extracellular IL4 signal, rapamycin complex 1 (mTORC1), known as the nutrition sensor mechanistic target, can be recruited from the cytosol to the lysosome membrane, and undergo phosphorylation (Sancak et al., 2010). In contrast, Lamtor1, the lysosome membrane-attached lysosomal adaptor protein complex regulator, together with its adjacent protein complex H^+^-ATPase (v-ATPase) responsible for integrating the extracellular signal with the intracellular nutrition sufficiency signal, are activated. An absence of Lamtor1 results in suppression of the combination of rapamycin complex 1 (mTORC1) in the lysosome membrane (Nada et al., 2009; Sancak et al., 2010; Bar-Peled et al., 2012). The cholesterol-sensing transcription factor liver X receptor (LXR) is thought to be one of the downstream targets of the amino acid-sensing pathway (v-ATPase, Lamtor1 and mTORC1) (Chantranupong et al., 2015; Kimura et al., 2016). Thus, Lamtor1, v-ATPase and mTORC1 integrate the intracellular amino acid sufficiency signal with the extracellular IL4 signal to control downstream LXR activity to produce the endogenous LXR ligand 25-hydroxycholesterol, which is ultimately associated with the expression of *Relma, Arg1* and *IL10* genes that characterize M2 macrophages (Figure 8B) (Kimura et al., 2016). We demonstrated that with the presence of similar amounts of IL4, A-ADM scaffolds and collagen both accelerated M2 polarization compared with the normal, and the absence of IL4 was the main cause leading to the failure of M2 polarization (Figures 4A–D). Down-regulation of Lamtor1 through siLamtor1 treatment resulted in significant regression of M2 polarization in those two groups (A-ADM^IL4+^ and collagen^IL4+^) to 3.71 and 3.69%, respectively, similar to the control^IL4+^ group with 3.46% (Figures 5B,C). We hypothesized that phagocytosis of the ADM scaffold-derived peptides by macrophages initiated the amino acid pathway. The intracellular amino acid sufficiency signal in the presence of IL4 regulated Lamtor1 and v-ATPase to integrate with mTORC1, resulting in the production of 25-hydroxycholesterol and subsequent activation of LXR and ultimately launching M2 polarization (Figure 8B) (Kimura et al., 2016).

Sadtler et el. demonstrated that when scaffolds were implanted into *RagI*^−/−^mice lacking T and B cells, scaffold-mediated IL4 up-regulation was lost and M2 polarization decreased, suggesting a T-cell-dependent T_H_2-driven scaffold immune microenvironment; they hypothesized that this process begins with an innate response during which the ECM component induces partial M2-like macrophage differentiation and simultaneously provide peptides to T cells, which together with IL4 production drives the T_H_2 response, significantly enhancing the M2 response (Sadtler et al., 2016). However, in our experiments, M2 macrophage polarization was significantly induced within 2 weeks, and the expression of the canonical T_H_2 cell gene (IL4) did not increase in parallel and levels similar to those in the saline groups were retained (Figures 2B,I,J). Further, with the presence of similar amounts of IL4, ADM scaffold- and collagen-treated macrophages *in vitro* also demonstrated enhanced M2 polarization, but knockdown of Lamtor1 reversed the increased M2 polarization (Figures 4D, 5C). Those results suggest that in contrast to the T_H_2-cell-induced pathway, the stably expression of IL4 does not directly affect macrophages during ADM scaffold-mediated induction of M2 polarization. Notably, activation of the amino acid-sensing pathway is also IL4 dependent (Kimura et al., 2016), which explains why in the *in vitro* experiments, additional IL4 was required for M2 macrophage polarization by the ADM scaffold and collagen. This result further demonstrates that macrophages are at least one of the cell types responsible for M2 polarization caused by the ADM scaffold and that this process involves the amino acid pathway.

Acellular dermal matrix-treated wounds showed enhanced vessel formation and hemorrhage avoidance in wound tissue formed at ~10 days of repair (Figures 1D,E). However, hemorrhage was observed in the saline-treated wounds at this early repair stage (Figures 1D, 7A). This phenomenon might be associated with the enhanced M2 polarization in the ADM-treated group. M2 macrophage signals have been widely reported to be related to collagen deposition and ECM morphology in tissues and organs (Wynn, 2004; Wynn and Ramalingam, 2012). In fact, the complex and multistep synthesis of collagen fibrils in embryonic development and tissue regeneration provides biochemical and functional stability in tissues and organs (Myllyharju and Kivirikko, 2004). Recent research has presented direct evidence that macrophage activation, particularly *Relm-*α (canonical M2 macrophage gene), is capable of inducing fibroblast production of lysyl hydroxylase 2 (LH2, encoded by the *Plod2* gene) (Knipper et al., 2015), a collagen-modifying enzyme. LH2 then directs dihydroxy lysinonorleucine (DHLNL) collagen cross-links, which determines the biochemical characteristics, matrix architecture, and mechanical properties of fibrillar collagens; their impact on tissue fragility or fibrosis in disease has been verified (Brinckmann et al., 2001; van der Slot et al., 2003; Myllyharju and Kivirikko, 2004; van der Slot-Verhoeven et al., 2005). Insufficient DHLNL cross-links will lead to defective structural and organizational stability of the ECM and further suppress the formation of vascular networks by disturbing endothelial-cell-matrix interactions (Davis and Senger, 2005). The disturbed endothelial-cell-matrix interactions may further result in a reduction in endothelial-tube-like structures and the acceleration of cluster assembly of endothelial cells in wound tissue (Malan et al., 2010, 2013). This cluster assembly of endothelial cells may be the dominant cause of the hemorrhage observed in saline-treated wounds (Figures 1D,E). In contrast, our results showed that in ADM-treated wounds, a critical canonical M2 macrophage gene, *Relm-a*, was highly up-regulated (Figure 2I). As the produced Relma is presented to fibroblasts, the increased LH2 induces sufficient DHLNL collagen cross-links, which orchestrate the collagen fibril structure and promote endothelial-cell fibril contacts, leading to well-organized tube formation, vascular integrity, and ultimately successful avoidance of hemorrhage in wound tissue (Figures 1D,E).

In most mammalian tissues, wound healing results in a scar, which is generally accompanied by the absence of subcutaneous fat. Scars with pathological fibrosis have been hypothesized to be caused by persistent activation or sustained mobilization of M2 macrophages (Spiller and Koh, 2017). Tgfβ is produced and activated by M2 macrophages and functions as a regulator to facilitate the resolution of inflammation; however, it also triggers fibroblast activation and conversion of ECM-producing myofibroblasts (Spiller and Koh, 2017). Myofibroblasts are a cell type that is present in many tissues, including the skin, liver, and nervous system, and they are mainly known to promote wound contraction, encourage collagen deposition, and secrete profibrotic cytokines (Eming et al., 2009; Gay et al., 2013). However, Plikus et al. observed the ability of myofibroblasts to convert into a completely different adipocyte lineage, and this unexpected conversion can aid scarless wound healing (Plikus et al., 2017). Adipose tissues have been shown to accelerate wound remodeling by mediating fibroblast migration and to further enhance hair follicle regeneration (Festa et al., 2011; Schmidt and Horsley, 2013). Our experimental results showed that the ADM scaffolds promoted adipose tissue formation in the newly reconstituted skin (Figure 7D). A similar phenomenon has been observed in hydrogel-treated acute skin injuries, in which the hydrogel promotes adipose tissue formation (Sun and Sun, 2017). Additionally, M2 polarization in ADM scaffold-treated wounds compared with that in saline-treated wounds greatly reduced the acceleration of fibrous encapsulation to approach normal collagen tissue (Figures 7B,E). Therefore, we speculate that ADM scaffolds might also have the potential in facilitating the conversion of myofibroblasts into adipocytes to reduce fibrotic tissue, which, certainly remains to be further explored. But if this is true, it will establish new insights into tissue regeneration via myofibroblast conversion intoL adipocytes.

## Ethics statement

All experimental protocols were approved by the Ethical Committee for Animal Experiments of South China Normal University. All animal experiments conducted in this research were performed in accordance with the guidelines of South China Normal University Intramural Animal Use and Care Committee and met the NIH guidelines for the care and use of laboratory animals.

## Author contributions

CH and XD designed and completed the study, and revised the manuscript. ZY was responsible for *in-vivo* experiment tissue harvest. YJ provided the main experimental instruments. XQ and JC performed the statistical analysis. All authors approved the manuscript.

### Conflict of interest statement

The authors declare that the research was conducted in the absence of any commercial or financial relationships that could be construed as a potential conflict of interest.
